# Comment on Hochfellner et al. Accuracy Assessment of the GlucoMen^®^ Day CGM System in Individuals with Type 1 Diabetes: A Pilot Study. *Biosensors* 2022, *12*, 106

**DOI:** 10.3390/bios13070709

**Published:** 2023-07-05

**Authors:** Guido Freckmann, Manuel Eichenlaub, Delia Waldenmaier, Stefan Pleus

**Affiliations:** Institut für Diabetes-Technologie, Forschungs-und Entwicklungsgesellschaft mbH an der Universität Ulm, D-89081 Ulm, Germany

In their recent article entitled “Accuracy Assessment of the GlucoMen^®^ Day CGM System in Individuals with Type 1 Diabetes: A Pilot Study” [1], Hochfellner and colleagues presented the results of an accuracy and usability evaluation of a novel continuous glucose monitoring (CGM) system. While we appreciate the importance of assessing the accuracy of CGM systems, we would like to comment on certain aspects of the data analysis, presentation of results and conclusions.

In the section describing the data analysis (Section 2.2), it is stated that “the lag time between CGM and blood glucose data was determined for each sensor and applied prior to calculating MARD and MAD”, where MARD and MAD are common accuracy parameters representing mean absolute relative and mean absolute deviation between CGM and comparator measurements, respectively. This correction of lag time before accuracy determination was common in microdialysis-based systems [2,3] or for prototype CGM systems [4]. However, in market-ready CGM systems intended to provide real-time glucose data such as the system examined in the article, the accuracy should be evaluated with respect to the CGM values displayed to the user. The effect of the lag time correction on the reported accuracy parameters cannot be assessed because neither the average lag time mentioned in Section 2.2. nor MARD and MAD results before lag time correction are presented. In general, however, a lag time correction leads to a reduction in MARD and MAD.

Regarding the MARD, it should be pointed out that for its calculation, only glucose levels ≥100 mg/dL were used (Table 1), whereas for glucose levels <100 mg/dL, MAD results were presented. This is an important detail missing from the abstract and the discussion, where MARD results were reported. 

As a consequence of choosing to correct for time lag and to use only glucose levels ≥100 mg/dL for MARD calculation, we would argue that the accuracy results reported in the article cannot be compared to the cited findings of CGM performance studies without lag time correction and MARD results determined across the full glucose range [5,6,7]. Another aspect of the reported study that impairs the comparability to previous results is the low sample size of eight subjects, which is not discussed. 

Due to these differences in methodology between this and other studies, we therefore question the validity of the conclusion that the “analysis suggests the GlucoMen^®^ Day CGM […] meets the current clinical requirements for state-of-the-art CGMs”.
